# 2021年非小细胞肺癌重要临床研究纵观与解读

**DOI:** 10.3779/j.issn.1009-3419.2022.101.19

**Published:** 2022-05-20

**Authors:** 彬 甘, 思阳 刘, 一龙 吴

**Affiliations:** 1 510080 广州，广东省肺癌研究所，广东省人民医院，广东省医学科学院 Lung Cancer Institute, Guangdong Provincial Key Laboratory of Translational Medicine in Lung Cancer, Guangdong Provincial People's Hospital, Guangdong Academy of Medical Sciences, Guangzhou 510080, China; 2 510632 广州，暨南大学附属第一医院血液科，暨南大学医学院血液学研究所，暨南大学教育部再生医学重点实验室 Department of Hematology, The First Affiliated Hospital of Jinan University; Hematology Institute, Jinan University Faculty of Medical Science; Key Laboratory for Regenerative Medicine, Ministry of Education, Guangzhou 510632, China

**Keywords:** 肺肿瘤, 辅助治疗, 靶向治疗, 免疫治疗, 个体化治疗, Lung neoplasms, Adjuvant therapy, Targeted therapy, Immunotherapy, Personalized medicine

## Abstract

尽管2021年新冠病毒仍在肆虐，非小细胞肺癌（non-small cell lung cancer, NSCLC）的临床研究并未止步。得益于肺癌治疗模式的进步，NSCLC患者的总生存时间和生活质量得到了显著改善。目前靶向治疗与免疫治疗的研究进展改变了术后辅助治疗的现状，建立了可手术NSCLC治疗新标准。局部晚期和晚期NSCLC同样有重要的研究进展，包括新的治疗模式、新的治疗药物等，均为临床治疗带来更多选择。相信在可预见的未来，这些疗法都将为NSCLC的治疗带来改变，并逐步走向肺癌“慢病化”的道路。因此，本文简要综述了2021年改变肺癌治疗临床实践的重要研究以及值得关注的研究进展。

## Abstract (en)

Though the coronavirus disease is still raging in 2021, clinical research on non-small cell lung cancer (NSCLC) did not stop. However, benefiting from advances in lung cancer treatment modality, NSCLC patients have experienced significant improvements in overall survival and quality of life. Currently, research advances on targeted therapy and immunotherapy have together transformed the status of postoperative adjuvant therapy and established a new standard treatment modality for resectable NSCLC. There are equally important research advances in locally advanced and advanced NSCLC, including new treatment modalities, new therapeutic agents, *etc*., all of which bringing more options for clinical treatment. These therapies will bring changes to NSCLC and will gradually lead to the chronicity of lung cancer in the foreseeable future. Therefore, this paper reviews important studies that will change clinical practice in NSCLC treatment and noteworthy research advances in 2021.

肺癌作为我国发生率和死亡率最高的恶性肿瘤^[1]^，一直是药物研究的攻克重点。近年来，精准治疗模式的飞速发展为患者带来切实的临床获益，在经历靶向治疗和免疫治疗两个重要的分水岭后，非小细胞肺癌（non-small cell lung cancer, NSCLC）的死亡率大大降低^[2]^。令人振奋的是，驱动基因阴性的程序性细胞死亡受体配体1（programmed cell death ligand 1, PD-L1）≥50%的晚期NSCLC患者的5年生存率相较于以往已经有了极大提升，为部分NSCLC患者带来长生存获益。得益于这些治疗手段的进步，尤其是创新药物和疗法的陆续获批上市，NSCLC患者的生存时间和生活质量得到了显著改善。2021年陆续有不少重磅研究值得我们共同关注，这些研究都为推动肺癌”慢病化“发展历程做出了贡献。

## 1 改变临床实践的重要研究

### 1.1 早期NSCLC的研究进展

早期NSCLC患者可以接受手术治疗，但仍有部分患者在术后会复发转移，病理分期为Ⅰb期/Ⅱa期/Ⅱb期/Ⅲa期的患者的5年总生存（overall survival, OS）率分别为68%、60%、53%和36%，Ⅲb期的患者则为26%^[3]^。因此患者术后获得长期生存至关重要的是降低肿瘤复发风险，尤其是远处复发。

#### 1.1.1 围手术期免疫治疗

以程序性死亡受体1（programmed death 1, PD-1）/PD-L1抑制剂为代表的免疫治疗已经改变了晚期NSCLC患者的治疗格局，而早期NSCLC患者机体免疫功能更好，理论上免疫治疗有望带来更好的疗效，因此免疫治疗关口前移、应用于早期可手术患者成为了近年来的研究热点。IMpower010和CheckMate 816两项Ⅲ期研究结果证实了免疫治疗在早期肺癌围手术期治疗中应用的前景。

IMpower010是一项全球多中心、开放标签、随机Ⅲ期研究，共入组1, 280例完全性切除的Ⅰb期（≥4 cm）-Ⅲa期[国际抗癌联盟（Union for International Cancer Control, UICC）第7版]NSCLC患者^[4]^。术后接受至多4个周期含铂化疗，1, 005例患者后续随机1:1分配，分别接受16个周期的阿替利珠单抗（1, 200 mg, *q21d*）或最佳支持治疗。主要研究终点为研究者评估的无病生存期（disease-free survival, DFS），次要终点包括OS。研究结果显示，对于完全切除的Ⅱ期-Ⅲa期NSCLC患者，辅助化疗后使用阿替利珠单抗相比最佳支持治疗显著改善了PD-L1表达≥1%患者的DFS。据此美国食品药品监督管理局（Food and Drug Administration, FDA）批准阿替利珠单抗用于PD-L1≥1%的Ⅱ期-Ⅲa期NSCLC的免疫辅助治疗。尽管IMpower010在PD-L1肿瘤阳性比例评分（tumor proportion score, TPS）≥1%的Ⅱ期-Ⅲa期人群中达到了主要终点，但DFS的改善主要由PD-L1 TPS≥50%的人群驱动（HR=0.43; 95%CI: 0.27-0.68），而在PD-L1 TPS 1%-49%的人群中，辅助免疫治疗的DFS获益有限（HR=0.87; 95%CI: 0.60-1.26）。尽管这种事后分析须谨慎看待，但这结果与过往转移性NSCLC的研究结果一致^[5, 6]^。因此临床实践可能更多用在PD-L1 TPS≥50%的Ⅱ期-Ⅲ期患者。此外值得注意的是，在IMpower010的复发模式分析中，免疫治疗没有影响局部或远处复发以及脑转移的发生率。因此免疫治疗在辅助治疗中到底起到什么作用、如何起作用还需要进一步的研究和探索。目前另一个Ⅲ期随机对照的辅助免疫治疗研究KEYNOTE-091^[7]^已宣布达到阳性结果，具体结果将在2022年的医学会议上公布，让我们拭目以待。

CheckMate 816是一项多中心、开放标签、随机Ⅲ期研究^[8]^，旨在评估相比传统标准治疗化疗，纳武利尤单抗联合化疗用于可切除NSCLC新辅助治疗是否可以改善患者生存结局的全球Ⅲ期临床研究。研究纳入358例Ⅰb期-Ⅲa期的可切除NSCLC患者，随机分为三个臂：①纳武利尤单抗联合化疗组；②单纯化疗组；③纳武利尤单抗联合伊匹木单抗组。研究的主要终点是BICR评估的完全病理缓解（complete pathological response, pCR）率和无事件生存期（event free survival, EFS）。结果显示，纳武利尤单抗联合化疗对比化疗可提高患者的pCR率和主要病理缓解（major pathologic response, MPR）率。另一研究终点EFS在年末时也已经提前宣布获得阳性结果。基于此项研究，FDA于2022年3月批准纳武利尤单抗联合化疗的NSCLC新辅助治疗适应证。

在IMpower010和CheckMate 816取得阳性结果后，便有个显而易见但仍无定论的问题需要解答：对于NSCLC患者，究竟应接受术后辅助免疫治疗还是新辅助免疫治疗？目前笔者认为对于具有一定手术难度的患者可考虑新辅助免疫治疗，而对于较易手术的患者可采取辅助免疫治疗。总体而言，新辅助免疫治疗似乎更容易被接受，期待未来能有更多研究和探索解答这个问题。

#### 1.1.2 术后辅助靶向治疗

表皮生长因子受体抑制剂（epidermal growth factor receptor tyrosine kinase inhibitor, EGFR-TKI）用于*EGFR*突变NSCLC患者术后辅助治疗的策略已纳入中国临床肿瘤学会（Chinese Society of Clinical Oncology, CSCO）、美国国立综合癌症网络（National Comprehensive Cancer Network, NCCN）等中外诊疗指南，无论是EVAN^[9]^、ADJUVANT/CTONG1104^[10]^、IMPACT^[11]^等一代EGFR-TKI的临床研究，还是ADAURA^[12]^等三代EGFR-TKI的临床试验，除IMPACT外，都显示了辅助靶向治疗为*EGFR*突变的NSCLC患者带来了良好获益。但在ADJUVANT/CTONG1104中可以看到，尽管吉非替尼辅助治疗*EGFR*突变的可切除Ⅱ期-Ⅲa期（N1-N2）NSCLC的无病生存期（disease-free survival, DFS）相对优于化疗（中位DFS：30.8 mon *vs* 19.8 mon；HR=0.56；95%CI：0.40-0.79；*P*=0.001）^[13]^。然而，并不是所有的患者都获得满意的临床结局，其OS并无显著差异，且部分患者出现复发，导致生存曲线有所下降，这表明有进一步生物标志物评估的必要性。

广东省人民医院吴一龙教授团队对ADJUVANT/CTONG1104临床研究进行的生物标志物探索性结果展示了一个新的评估模型^[14]^。该研究人群为随机入组ADJUVANT/CTONG1104的Ⅱ期-Ⅲa期（N1-N2）、*EGFR*突变型NSCLC、接受了完全手术切除的其中171例患者，对其组织进行422个癌症相关基因分析，结合DFS识别预测性生物标志物，进一步探索对于*EGFR*突变的患者存在哪些预测辅助治疗疗效的基因变异。结果显示，携带*NKX2-1*、*CDK4*和*MYC*扩增、*TP53*外显子4/5错义突变的*EGFR*突变患者术后辅助TKI的生存相对更好，而*RB1*变异的患者辅助化疗相对更好。研究者据此构建了MINERVA评分模型（Multi-gene INdex to Evaluate the Relative benefit of Various Adjuvant therapies），能综合评估肿瘤的个体化治疗反应。通过该模型评分能将患者分为三组：TKI高度优先组（HTP）患者：辅助吉非替尼治疗无论DFS和OS均具有显著优势，亚组特征为富集了*NKX2-1*、*CDK4*和*MYC*扩增、*TP53*外显子4/5错义突变；TKI优先组（TP）患者：辅助吉非替尼DFS具有显著优势，缺乏富集的预测性生物标志物；化疗优先组（CP）：患者尽管携带*EGFR*突变，但对化疗反应更好且有DFS获益，其亚组特征为存在*RB1*变异。利用Ⅱ期新辅助研究CTONG1103的数据进一步验证该模型，观察到类似的预后分层结果。MINERVA通过生物标志物对患者进行分组，让精准治疗更精准，为个性化辅助治疗发展提供了新视角。

结合目前已有的临床研究证据^[9-11]^，一代EGFR-TKI用作NSCLC辅助靶向治疗的临床结局几乎相同：用药两年后停药，仍可以持续获益2年。而且，在一项真实世界研究^[15]^中显示，无论*EGFR*突变状态和术后阶段如何，辅助化疗并没有改善生存结果，提示NSCLC术后辅助化疗似乎不是必须。当然还需要思考一代EGFR-TKI在辅助治疗中的作用，例如一代和三代EGFR-TKI的最佳序贯治疗策略、治疗时间、耐药机制以及疾病复发后的后续治疗；生物标志物和微小病灶残留（minimal residual disease, MRD）可能是突破目前困境的潜在途径，指导辅助靶向治疗，使患者的生存获益最大化。

目前早期NSCLC的多学科治疗模式可总结如下：对于Ⅰa期的患者，手术是最优选择，部分情况可选择体部立体定向放射治疗（stereotactic body radiotherapy, SBRT）；对于可手术的Ⅰb期-Ⅲ期患者，术后需检测*EGFR*基因突变和PD-L1蛋白表达两个指标，根据检测结果，若为*EGFR*阳性则可采用辅助靶向治疗，若为PD-L1阳性则可考虑在辅助化疗后增加免疫巩固治疗；对于有潜在手术可能的Ⅲ期患者，根据检测结果可考虑术前进行新辅助治疗。

### 1.2 局部晚期NSCLC的研究进展

25%-30%的NSCLC患者诊断时便处于不可手术的Ⅲa期-Ⅲc期^[16]^。过往Ⅲ期NSCLC的标准治疗只有同步放化疗（concurrent chemoradiotherapy, cCRT）^[17]^，且研究进展缓慢。PACIFIC研究^[18]^结果在公布之初被称为“海啸”，首次证实了同步放化疗后加入免疫巩固治疗可显著改善不可切除的Ⅲ期NSCLC患者的生存，随之便改变了临床实践进入标准治疗。近期更新的5年生存率达到42.9%，切实为患者带来显著的生存获益^[19]^。然而在真实世界中，由于同步放化疗的毒性较大，很多患者都无法耐受^[20]^。在中国多数会采取序贯放化疗（sequential chemoradiotherapy, sCRT）方案作为替代，因此这部分患者的免疫巩固治疗亟需临床证据支持。

GEMSTONE-301是一项随机双盲、安慰剂对照的Ⅲ期研究^[21]^，旨在评估舒格利单抗作为巩固治疗在同步或序贯放化疗后未发生疾病进展的、不可切除的Ⅲ期NSCLC患者中的有效性和安全性。研究共纳入来自50家中心的381例患者，其中33.3%的患者之前接受序贯放化疗，69.6%的患者美国东部肿瘤协作组（Eastern Cooperative Oncology Group, ECOG）体力状况评分为1分，69.0%的患者为鳞状细胞癌，Ⅲa期、Ⅲb期、Ⅲc期患者分别占28%、55%、16%。患者按2:1随机接受舒格利单抗或安慰剂巩固治疗。研究结果显示，舒格利单抗组相较于安慰剂组能显著延长无进展生存期（progression-free survival, PFS）（9.0 mon *vs* 5.8 mon; HR=0.64, 95%CI: 0.48-0.85, *P*=0.002, 6）。而且，无论同步还是序贯放化疗后的患者均显示出临床获益。

GEMSTONE-301研究扩展了PACIFIC研究的入组人群，在PACIFIC模式基础上增加对sCRT的评估，使其治疗模式能涵盖超过90%的Ⅲ期NSCLC患者。鉴于东亚人群中*EGFR*突变率更高，且免疫治疗对*EGFR/ALK/ROS1*突变人群的作用极为有限，研究明确排除了驱动基因阳性的患者，极大程度减少了基因突变异质性对治疗效果的影响。尽管GEMSTONE-301相较于PACIFIC的Ⅲb期、Ⅲc期患者比例更高、鳞癌患者更多，但关键结果非常接近，同时也为序贯放化疗后接受免疫巩固治疗能获益提供了坚实的临床证据。

近年来，免疫治疗在局部晚期NSCLC领域除了免疫巩固治疗的不断探索，还有免疫同步放化疗的研究。真实世界调查^[22, 23]^显示，接受cCRT的不可切除Ⅲ期NSCLC患者中，22%-30%患者因出现进展或不能耐受毒性而无法进行免疫维持治疗。开放标签、非随机、全球多中心的Ⅱ期研究KEYNOTE-799^[24]^将免疫治疗的时机提前，共纳入216例初治不可切除Ⅲa期‒Ⅲc期NSCLC患者，其中112例患者进入队列A（鳞癌和非鳞癌NSCLC），104例患者进入队列B（非鳞癌NSCLC），在同步放化疗期间进行2或3周期帕博利珠单抗治疗，后续给予14个周期免疫治疗。主要研究终点为客观缓解率（objective response rate, ORR）和3级以上间质性肺炎发生率。研究结果显示这种模式有一定获益，ORR约为70%，提示帕博利珠单抗同步cCRT对Ⅲ期不可切NSCLC有良好的抗肿瘤疗效，期待进一步的Ⅲ期临床研究验证结果。

目前有不少针对Ⅲ期NSCLC的免疫治疗与放化疗联合的大型临床研究正在开展，如PACIFIC 2、PACIFIC 5、SKYSCRAPER-3、CheckMate 73L等，但研究设计均较为保守，希望有更具突破性的研究能颠覆当前的治疗模式，为患者带来更显著的生存获益提升。

目前不可手术局部晚期NSCLC的多学科治疗模式可总结如下：对于Ⅲ期患者，同样需要检测*EGFR*基因突变和PD-L1蛋白表达两个指标，若为*EGFR*阳性的患者，不主张选择包含免疫治疗的方案；若为PD-L1阳性的患者，无论同步还是序贯放化疗后，建议考虑采用免疫巩固治疗。

### 1.3 晚期NSCLC的研究进展

抗体偶联药物（antibody-drug conjugate, ADC）是一种能够将细胞毒性药物直接递送至肿瘤细胞的抗肿瘤治疗方式，由三部分组成：细胞毒素（payload）、单克隆抗体（mAb）与连接子（Linker）^[25]^。主要机制为抗体结合靶抗原、内化后连接子断裂，释放细胞毒素，最终达到肿瘤杀伤最大化以及全身毒性最小化的目的^[26]^。

目前ADC在肺癌治疗领域的研发非常火热，尤其是应用于HER2靶点。Trastuzumab Deruxtecan（DS-8201）是一种新出现、靶向HER2的ADC，由人源化抗HER2 IgG1单抗（曲妥珠单抗）与拓扑异构酶I抑制剂DXd通过可裂解的四肽连接子连接而成。DESTINY-Lung01是一项全球多中心的Ⅱ期研究^[27]^，旨在评估DS-8201治疗*HER2*过表达/突变的晚期NSCLC疗效。研究入组了91例携带*HER2*突变的晚期非鳞肺癌患者（90例既往接受过抗肿瘤治疗），接受DS-8201 6.4 mg/kg，每3周1次。主要研究终点是ICR评估的客观缓解率（objective response rate, ORR）。研究结果显示，DS-8021用于经治*HER2*突变晚期NSCLC的ORR为55%，OS为17.8个月。与之对照的是，同样报道于ESMO 2021的ZENITH20-4研究^[28]^，小分子TKI波奇替尼（Poziotinib）的一线ORR为43.8%，与ADC药物的数据相比逊色不少。但是不是就意味着ADC就全面占优呢？事实并非如此，我们需要注意到DS-8201的安全性问题，3级以上的药物相关不良事件占46%，5级致死性不良事件则达14.3%，均提示DS-8201的安全性需要进一步验证。

在ADC仍在探索的阶段，被视为下一代ADC的免疫刺激偶联药物（immune stimulating antibody conjugate, ISAC）已经出发^[29]^。BDC-1001由HER2靶向的曲妥珠单抗生物类似物与TLR7/8激动剂偶联而来，通过曲妥珠单抗介导的机制直接杀死肿瘤细胞，激活的髓系APC局部吞噬和清除HER2表达的肿瘤细胞，以及T细胞对肿瘤相关抗原或新抗原的持久的免疫反应发挥抗肿瘤活性。可惜临床结果令人失望，BDC-1001治疗的40例可评估患者中，ORR仅为2.5%，疾病控制率（disease control rate, DCR）为32.5%^[30]^。更可惜的是这并非孤例，同期报道于ESMO-IO 2021的ISAC SBT6050的Ⅰ期临床数据显示，其ORR仅为7%^[31]^。可见，联合细胞毒素的ADC是任重道远，而其他的联合方案是困难重重。

晚期NSCLC值得关注的另一研究是ORIENT-31^[32]^。过往*EGFR*突变的NSCLC患者被认为无法从免疫治疗中获益^[33]^，但在IMpower150亚组分析发现^[34]^，阿替利珠单抗联合贝伐珠单抗及化疗相较于贝伐珠单抗联合化疗可以改善*EGFR*突变人群的OS，但样本量较小且为亚组分析，需前瞻性大样本临床研究进一步验证。ORIENT-31是一项随机双盲Ⅲ期临床研究，旨在评估信迪利单抗联合贝伐珠单抗及化疗对EGFR-TKI治疗失败的非鳞状NSCLC的疗效与安全性。首次中期分析显示，与单纯化疗组相比，信迪利单抗的四药组合显著延长中位PFS（6.9 mon *vs* 4.3 mon; HR=0.464, 95%CI: 0.337-0.639; *P* < 0.000, 1）。期待该研究报道更多成熟的数据，推动临床实践的改变。

## 2 重要的阴性结果研究

往往我们大家以为阳性结果的研究才会改变临床决策，但其实部分阴性结果同样能产生重要影响。

奥希替尼不宜与贝伐珠单抗一线联合使用：WJOG 9717L是一项开放标签随机Ⅱ期试验^[35]^，该研究旨在比较奥希替尼联合贝伐珠单抗对比奥希替尼单药治疗初治晚期*EGFR*突变非鳞NSCLC患者的临床疗效。结果显示相比奥希替尼单药治疗，奥希替尼与贝伐珠单抗联用后，未能改善mPFS（22.1 mon *vs* 20.2 mon; HR=0.862, 95%CI: 0.531-1.397; *P*=0.213），且显著增加不良反应，尤其是高血压（8.3% *vs* 34.4%）、蛋白尿（6.7% *vs* 54.1%）、鼻血（8.3% *vs* 36.1%），提示奥希替尼不宜与贝伐珠单抗一线联合使用。

纳武利尤单抗不宜用于EGFR-TKI耐药后治疗：WJOG 8515L研究是一项多中心随机Ⅱ期研究^[36]^，其评估了纳武利尤单抗对比卡铂联合培美曲塞用于EGFR-TKI治疗失败的EGFR阳性NSCLC患者的疗效。主要研究终点结果显示，纳武利尤单抗组的mPFS差于化疗组（1.7 mon *vs* 5.6 mon; HR=1.92, 95%CI: 1.27-2.90; *P*=0.008），提示免疫单药治疗无法应用于EGFR-TKI耐药后的治疗。

免疫联合抗血管药物一线治疗应慎重：Ⅲ期研究LEAP-007采取随机双盲的临床设计，入组既往未经治疗的PD-L1 TPS≥1%的无*EGFR*、*ROS1*或*ALK*突变的Ⅳ期NSCLC患者，旨在进一步探索PD-L1阳性晚期NSCLC患者一线接受帕博利珠单抗±仑伐替尼治疗的临床获益^[37]^。结果表明，尽管PFS和ORR有改善，但该联合模式未能为患者带来OS的获益。大相径庭的是，信迪利单抗联合安罗替尼的Ⅰb期研究结果显示出良好信号，ORR达72.7%，mPFS达15个月^[38]^。而Sebastian教授等^[39]^则在同期评述中起了《Too Good to be True?》的题目表达疑虑。因此目前对于免疫联合抗血管药物应用在一线治疗NSCLC应该慎重，需要更多的大型临床证据来验证。

Canakinumab治疗肺癌前景堪忧：Canakinumab是一种人源单克隆抗体，与人白细胞介素-1β（interleukin-1β, IL-1β）具有高亲和力和选择性结合，并通过阻断其与受体的相互作用来中和IL-1β的活性，从而抑制促肿瘤炎症（pro-tumor inflammation, PTI），增强抗肿瘤免疫反应，达到抗肿瘤目的。Ⅲ期研究CANOPY-1^[40]^旨在评估Canakinumab联合帕博利珠单抗和含铂双药化疗一线治疗晚期NSCLC的疗效，结果显示未能达到OS和PFS共同研究终点。评估Canakinumab联合多西他赛二、三线治疗的Ⅲ期CANOPY-2^[41]^也同样失败。这些结果无法验证PTI抗肿瘤的概念，应评估这类药物的发展和应用前景。

## 3 值得关注的研究

COAST研究（开放标签随机Ⅱ期）在PACIFIC模式上继续创新，以PACIFIC模式作为对照组，评估度伐利尤单抗联合新药Oleclumab或Monalizumab的疗效^[42]^。正常情况下，放疗会诱导CD73和HLA-E（NKG2A配体）的表达，从而抑制抗肿瘤免疫反应^[43-46]^。而Oleclumab可以抑制CD73以减少细胞外腺苷的产生，从而促进抗肿瘤免疫力^[47]^。Monalizumab可以阻断NKG2A以减少对自然杀伤（natural killer, NK）细胞和CD8^+^ T细胞的抑制^[48]^。该研究设计从免疫循环的整体角度入手^[49]^，寻找合适的联合治疗模式，是非常好的创新思路。研究结果初步验证了理论假设，无论ORR还是mPFS都有显著提升，期待下一次研究结果公布。

TACTI-002（开放标签Ⅱ期）显示出良好的临床信号，帕博利珠单抗联合新药Eftilagimod alpha（可溶性LAG-3蛋白）一线治疗PD-L1表达未经选择的晚期NSCLC，mPFS达8.2个月，对于PD-L1 TPS≥50%的患者ORR为54%^[50]^。该方案已在黑色素瘤中取得成功^[51]^，期待肺癌领域的进一步研究。

Ⅲ期ATALANTE-1研究^[52]^展示了治疗性肿瘤疫苗的临床应用价值，结果显示在晚期HLA-A2^+^ NSCLC患者中，OSE2101与化疗相比具有良好的临床获益。对于免疫治疗耐药的患者，OS显著提升，死亡风险下降（11.1 mon *vs* 7.5 mon; HR=0.59, 95%CI: 0.38-0.91; *P*=0.017），提示疫苗正在走进临床实践。

ROS1抑制剂Entrectinib治疗*ROS1*融合阳性NSCLC疗效显著，合并分析ALKA-372-001、STARTRK-1、STARTRK-2三个Ⅰ期/Ⅱ期的结果^[53]^显示，ORR达67.1%，其中颅内控制率达79.2%，希望这令人振奋的疗效能很快进入临床实践。

DZD9008（选择性、不可逆的EGFR-TKI）在既往接受过治疗的*EGFR*外显子20插入突变的NSCLC患者中显示了良好的抗肿瘤疗效，在200 mg/d和300 mg/d剂量下，ORR分别达到45.5%和41.9%^[54]^。*EGFR*外显子20突变的异质性很强，可细分为近端（near-loop）和远端（far-loop）两个亚组，其对EGFR-TKI的敏感性也不相同^[55]^。而DZD9008在近端的ORR为47.5%，在远端的ORR为36.4%。基于此，美国FDA授予DZD9008突破性疗法认定，用于治疗携带*EGFR*外显子20插入突变的NSCLC患者。后续的研究也值得继续关注。

Ⅰ期CHRYSALIS研究评估了双特异性抗体Amivantamab单药、Amivantamab+Lazertinib用于三代TKI耐药的*EGFR*突变NSCLC的安全性和疗效，结果显示ORR为36%，中位缓解持续时间（duration of overall response, DOR）为9.6个月^[56, 57]^。Ⅰ期CHRYSALIS-2研究^[58]^评估了Lazertinib单药疗法及与Amivantamab联合用药治疗晚期NSCLC患者，在既往接受了2线-3线治疗的29例患者中ORR为41%，既往接受了4线及以上治疗的47例患者中ORR为21%。为三代EGFR-TKI耐药进展后的患者提供了新的治疗希望，这种治疗方案或能进一步延长患者的生存获益，期待有更多样本量的临床研究验证。

## 4 回顾与展望

回顾2021年值得关注的重要研究，首先，可以发现最显著的是早期NSCLC的研究进展，靶向辅助治疗彻底改变了整个治疗模式，并且是“精准再精准”，为个体化治疗提供了全新视角；免疫辅助治疗同样亮眼，不仅能应用于术后辅助治疗，而且还能应用于序贯放化疗后的巩固治疗，对于可手术NSCLC，靶向或免疫的辅助治疗将成为标准；其次，对于局部晚期NSCLC，序贯放化疗后免疫治疗已经验证可减少疾病复发，为临床和患者带来更多治疗选择；再次，晚期NSCLC的研究进展颇多，其中抗体偶联药物（antibody-drug conjugate, ADC）作为突破现有治疗机制的药物可能会成为新的治疗手段；最后，免疫检查点抑制剂的研究继续如火如荼，在治疗驱动基因阳性NSCLC方面已经初现曙光。我们共同期待未来能有更多更坚实的临床证据涌现，有更多更好的治疗药物用于临床实践，为NSCLC治疗带来更多变革，造福患者。
